# Antibody-Based Immunotherapeutic Strategies for COVID-19

**DOI:** 10.3390/pathogens9110917

**Published:** 2020-11-05

**Authors:** Jamal Hussen, Mahmoud Kandeel, Maged Gomaa Hemida, Abdullah I. A. Al-Mubarak

**Affiliations:** 1 Department of Microbiology, College of Veterinary Medicine, King Faisal University, Al-Ahsa 31982, Saudi Arabia; jhussen@kfu.edu.sa (J.H.); mhemida@kfu.edu.sa (M.G.H.); 2 Department of Biomedical Sciences, College of Veterinary Medicine, King Faisal University, Al-Hofuf, Al-Ahsa 31982, Saudi Arabia; mkandeel@kfu.edu.sa; 3 Department of Pharmacology, Faculty of Veterinary Medicine, Kafrelshikh University, Kafrelshikh 33516, Egypt; 4 Department of Virology, Faculty of Veterinary Medicine, Kafrelshikh University, Kafrelshikh 33516, Egypt

**Keywords:** SARS CoV-2, COVID-19, immunotherapy, clinical trials, antibodies

## Abstract

Global efforts to contain the coronavirus disease-2019 (COVID-19) include the development of novel preventive vaccines and effective therapeutics. Passive antibody therapies using convalescent plasma, SARS-CoV-2 (Severe-Acute-Respiratory-Syndrome-Corona-Virus-2)-specific neutralizing antibodies (NAbs), and the development of monoclonal antibodies (MAbs) are among the most promising strategies for prophylaxis and treatment of SARS-CoV-2 infections. In addition, several immunomodulatory antibodies acting via several mechanisms to boost the host immune defense against SARS-CoV-2 infection as well as to avoid the harmful overreaction of the immune system are currently under clinical trial. Our main objective is to present the current most up-to-date progress in some clinical trials registered at ClinicalTrials.gov. We highlight the pros and pitfalls of several SARS-CoV-2 antibody-based immunotherapeutics.

## 1. Introduction

SARS-CoV-2 is the third zoonotic coronavirus that emerged in the last decade after the SARS-CoV in 2003 and the Middle East respiratory syndrome coronavirus (MERS-CoV) in 2012 [1,2]. They have the criteria of a high rate of infection and spread among a close contact population [3,4,5,6]. This high rate of transmission worldwide contributed to the development of the current pandemic sweeping the globe. Although the majority of individuals with COVID-19 exhibit only mild-to-moderate symptoms, about 15% of infected people have a progressive course of infection. Some of these cases develop a severe form of the disease characterized by acute respiratory distress syndrome (ARDS) and septic shock [1,2,7,8,9,10,11]. Similar to other coronaviruses, SARS-CoV-2 uses its spike (S) protein for receptor binding and virus entry into the target cells [12,13,14,15,16,17,18]. Several recent studies demonstrated that both SARS-CoV and SARS-CoV-2 use the same receptor, the angiotensin-converting enzyme 2 (ACE2), for cell entry [17,18,19].

**Figure 1:**
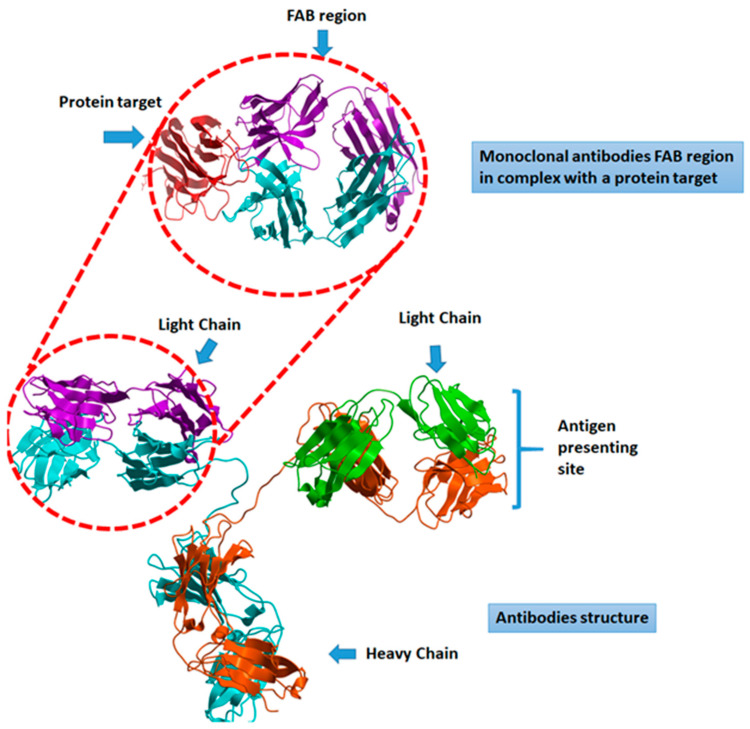
The structure of antibodies showing the interaction of FAB (Fragment Antigen Binding) with a target protein.

Looking for effective treatments for COVID-19, there is increasing interest in antibody-based immunotherapeutics such as convalescent plasma, neutralizing antibodies (NAbs), monoclonal antibodies (MAbs), and intravenous immunoglobulins (IVIg). In this context, a highly specific antibody can be generated for targeting both host and viral target proteins (Figure 1).

There are several monoclonal antibody-based treatments under planned clinical trials. More than 30 trials are currently taking place mostly in the U.S., China, and Europe, evaluating the use of mAb therapies for COVID-19. The current study aims to review the main clinical trials registered on ClinicalTrials.gov for the use of antibody-based immunotherapeutics for prophylactic and therapeutic purposes against SARSCoV2 infection.

## 2. Some Recent Findings on the Immunobiology of SARS-CoV-2 Infection

Recent research shows that SARS-CoV-2 infection triggers the induction of both innate and adaptive immune responses by the infected person, which play essential roles in the elimination of the viral infection [20,21,22,23,24,25,26,27,28]. However, the over-activated innate immune responses and impaired adaptive immune responses may result in immunopathology leading to severe local and systemic tissue damage in the patient [29,30,31,32,33]. The process of SARS-CoV-2 entry into the cells involves the participation of several key proteins from both the virus and the host cells. Simply, SARS-CoV-2 enters the host cells, mainly after binding to the ACE-2 receptors that are expressed by some target cells [34]. In addition, the role of the C-type lectin L-SIGN, which is expressed in human lung alveolar epithelial type II cells, in mediating SARS-CoV-2 entry to the host cell has also been recently reported [21]. The activation of the innate immune response against SARS-CoV-2 infection is mediated by the interaction of the pathogen-associated molecular patterns (PAMPs) from the virus side and the pattern recognition receptors (PRRs) of innate immune host cells. In addition to the toll-like receptors (TLRs), the PRRs include the nucleotide-binding and oligomerization domain (NOD)-like receptors (NLRs) and the retinoic acid-inducible gene I (RIG-I)-like receptors (RLRs) [20]. The interaction between both the PRR and PAMP leads to the activation of intracellular signaling pathways in the epithelial cells of the innate immune cells of the respiratory tract, including alveolar macrophages, neutrophils, monocytes, and natural killer (NK) cells. The stimulated cells produce several immune mediators, including the type-I-IFN, (IFNα/β) and type II IFN (IFNγ), inflammatory cytokines (IL-6 and IL-1β), as well as some chemokines such as CXCL-10 and CCL-2 [35,36]. The innate immune cells, especially macrophages and dendritic cells, play essential roles in mounting the adaptive immune response by presenting antigens to the helper T cells. The development of the protective adaptive immunity to SARS-CoV-2 infection mainly depends on the activation of both humoral and cell-mediated immune responses. Thus, the activation of the CD4-positive T helper cells, which help the B-cells in the process of the production of specific neutralizing antibodies. Meanwhile, activation of the cytotoxic CD8-positive T cells results in the effective elimination of infected cells [37]. In COVID-19 patients, cytotoxic T cells account for about 80% of the cell population infiltrating the lung-tissue [35]. Typically, the process of the B cell activation and the production of virus-specific antibodies is an essential event for controlling most viral diseases, including COVID-19. The process of cross-bridging of two or more B cell receptors (surface immunoglobulins) by viral antigenic epitopes together with co-activation through the activated helper T cells results in the formation of plasma and memory B cells. This process triggers the production of various isotypes of virus-specific antibodies. The mechanisms of antibody-mediated protection against viral infection include several processes, including virus neutralization, virus opsonization, phagocytosis, and NK-cell-mediated elimination of virus-infected cells by antibody-dependent cellular cytotoxicity (ADCC) [20]. The recovery of some patients with SARS-COV-2 was associated with the production of SARS-CoV-2-specific antibodies [3]. In addition, the protective role of SARS-CoV-2-neutralizing antibodies has been demonstrated by blocking the experimental infection in some animal models [21]. Recent studies show that SARS-CoV-2 infection activates the humoral immune response, which triggers the production of different isotypes of virus-specific antibodies. The production of the antibody-producing plasma cells (ASCs) has been shown to increase substantially during SARS-CoV-2 infection [38]. The detection curve of the SARS-CoV-2-specific antibodies after the onset of the clinical signs in the affected patients varies according to the type of antibodies. Both IgA and IgM are still detectable from two to six days of the onset of the clinical symptoms, while the IgG is still detectable from 10 to 18 days. A recent study investigated the seropositivity rate for IgG and IgM antibodies in some COVID-19 patients 14 days after the onset of symptoms. This study found higher titers of antibodies against the surface spike protein receptor-binding domain (RBD) compared to the internal nucleocapsid protein (NP) antigen. [39]. The same study revealed a correlation between the titer of antibodies against NP and RBD and their neutralizing effect.

## 3. Some Putative Mechanisms for the Immunopathology of SARS-CoV2 Infection

The dysregulated immune responses to viral infections, which fail to stop viral replication and to eliminate infected cells, may result in a hyperinflammatory response. This pattern of responses leads to the uncontrolled release of pro-inflammatory cytokines (cytokine storm) and the development of systemic inflammation and multi-organ failure [40]. The cytokine storm syndrome has been documented in several viral diseases and is characterized by the uncontrolled host immune defense and the massive release of pro-inflammatory cytokines and chemokines from the neutrophils and monocytes [40]. Several recent studies provide evidence that cytokine storm contributes substantially to the severe acute respiratory distress syndrome (ARDS) and respiratory failure in patients with severe COVID-19 [41,42,43]. A recent study analyzed the expression levels of several immune mediators, including many cytokines and chemokines in plasma of 150 COVID-19 cases in Wuhan. The same study showed significant elevation in the expression levels of the cytokines IL-1β, IL-1Rα, IL-7, IL-8, IL-9, IL-10, IFN-γ, and TNFα, and the chemokines MIP1α (macrophage inflammatory protein 1-alpha), MIP1β, MCP1 (monocyte chemoattractant protein 1), and IP10 (interferon gamma-induced protein 10), compared to non-infected healthy individuals [44]. The same study found a significant increase in the IL-6 levels among dead patients compared with the survivors [44]. Another recent study reported higher levels of TNF-α, IL-2, IL-7, GCSF, IP10, MCP1, and MIP1α, in cases with sever disease compared to the mild COVID-19 cases [43]. The production and recruitment of massive amounts of inflammatory cytokines, such as IL-1β, IL-6, and TNF-α, enhances the influx of additional inflammatory immune cells such as neutrophils and monocytes to the site of infection. This process may lead to tissue damage in several vital organs (lungs, heart, liver, and kidneys), resulting in respiratory failure or multiple organ failure in many cases [43].

The main cellular immunopathologic markers for the severe COVID-19 cases are a marked reduction in the number of monocytes as well as lymphocytopenia in addition to a substantial decrease in the numbers of all circulating lymphocyte subsets, including CD4^+^ T cells, CD8^+^ T cells, B cells, and natural killer (NK) cells. On the other hand, the numbers of CD4+ T cells, CD8+ T cells, B cells, and NK cells normalize in patients who have recovered from the COVID-19 [45,46]. The binding of surface molecules involved in T cell activation, especially CD26 and CD147, to the SARS-CoV-2-S protein contribute to the lymphopenia reported in COVID-19-patients through activation-induced T cell death [37]. Regarding the granulocyte population during the course of SARS-CoV-2 infection, the numbers of eosinophils and basophils were decreased, while the number of neutrophils was reduced. During SARS-CoV-2 infection, there is an association between the increased lymphocytes numbers and the decreased neutrophils numbers. An increase in the neutrophil to lymphocyte ratio (NLR) has been linked to severe SARS-CoV-2 infections [18,38,47]. The role of the humoral immune response in the immunopathology of SARS-CoV-2 infection has been recently investigated. Due to the link between increased patient IgG response and the worse outcome of COVID-19, a possible role of antibody-dependent enhancement (ADE) of SARS-CoV-2 infection has been suggested [48,49]. 

## 4. The Antibody-Based Therapies for SARS-CoV-2 Infections

Due to the lack of any specific drugs or vaccines for COVID-19 infections to date, researchers around the world are currently working on testing some potential vaccines and effective therapies to stop the spreading of this pandemic and to contain this virus. In a pandemic situation such as COVID-19, active immunization against SARS-CoV-2 is one of the best control remedies. However, vaccine development is a complicated and time-consuming procedure, which may take a long time for approval and availability in the commercial market. To bridge the gap resulting from the lack of an efficient vaccine against SARS-CoV-2, the short-term immunity induced by using the antibody-based immunotherapeutic strategies represents an effective alternative. The main advantage of this approach is the shorter timeline from development and testing to approval compared to vaccines or other chemical drugs. Antibody-based immunotherapeutics such as convalescent plasma, NAbs, MAbs, and IVIg have been in use for decades and have a proven record of safety and efficacy [50]. Some of them have been found to be useful in managing COVID-19 patients [51].

The development of effective antibody-based immunotherapeutics against COVID-19 is less time-consuming when compared with the development of new vaccines. Antibody-based therapies also provide an alternative method for the prevention or treatment of COVID-19 in special cases, where vaccination may not lead to mounting protective immune responses, such as elderly and immune-compromised individuals.

There are different antibody-based immunotherapeutic approaches, which are currently investigated for the treatment and prevention of COVID-19. These approaches are mainly based on two strategies, which are the employment of SARS-CoV-2-neutralizing antibodies and the immunomodulatory antibodies, which act via several mechanisms to support the immune defense against the virus or to avoid the life-threatening overreaction of the immune system. 

### 4.1. Antiviral Neutralizing Antibody-Based Therapeutics

The correlation between the levels of antibodies detected in some COVID-19 patients and a virus neutralization effect opened the possibility of using convalescent plasma from the COVID-19 survivors for the treatment of some patients with SARS-CoV-2, especially in severe cases [52]. The passive antibody therapy using convalescent plasma involves the administration of the acellular portion of the blood from the recovered patients to the individuals who are infected or at risk of infection [52,53]. In a recent study involving individuals with severe cases of COVID-19, the administration of plasma from recovered patients resulted in a rapid increase in the level of the serum-neutralizing antibody titers in the recipients [54]. This was in association with a reduction in total viral load and better outcomes in the affected patients received this treatment [54]. A significant clinical improvement of some COVID-19 patients after the transfusion of convalescent sera with no detectable SARS-CoV-2 viral RNA in their blood has also been reported in another clinical trial [55]. Although it seems promising for the treatment of COVID-19, this approach is associated with several limitations. The batch variability (plasma from different individuals may contain different mixtures of antibodies with different virus-neutralizing capacities) and the need for blood type matching and screening for blood-borne pathogens, including HIV and hepatitis viruses, are major limitations for using the convalescent plasma for the treatment of COVID-19. The highly specific antiviral monoclonal antibodies are currently suggested as an alternative to plasma therapy. The SARS-CoV-2-S protein is mainly responsible for binding on cell surface receptors—the ACE2—and for the fusion with the host cell membrane and thus, it represents the main target for neutralizing monoclonal antibody therapy [52]. Therefore, several therapeutic monoclonal antibodies specific to SARS-CoV-2 are currently under clinical trials. The antiviral monoclonal antibodies can be recovered by several techniques, including yeast- or phage display-based in vitro selection approaches, antibody production in animals followed by antibody humanization, and the sorting of a single antigen-specific B cell [52]. The potential uses of the monoclonal antibodies in the clinical applications against COVID-19 depend on several characteristics, including their SARS-CoV-2-neutralizing ability, the type of the target SARS-CoV-2-S protein epitopes, and their Fc-portion-mediated effector functions. Therefore, several immunoglobulin engineering techniques are currently used for optimizing the outcomes and the pharmacokinetics of SARS-CoV-2 monoclonal antibody therapeutics, including selective isotype switching, the Fc modifications by the substitutions of distinct glycans or amino acids that modify the Fc region affinity for the Fc receptors [52]. These modifications may also help in avoiding undesirable effects of COVID-19 monoclonal antibodies through the antibody-dependent enhancement (ADE) infection of the immune cells, including monocytes, macrophages, and B cells, which has been reported for coronaviruses [52]. Another approach was adopted for reducing the cost of the monoclonal antibody production. This is through the administration of the DNA or the messenger RNA (mRNA) encoding the desired antibody to humans. This approach allows their production in vivo instead of ex vivo [56]. Due to their effective virus neutralization potential, mAbs against the RBD of the S1 subunit of SARS-CoV-2-S protein are currently targeted in several clinical trials aiming for the development of new mAbs and the application of the existing SARS-CoV-2-specific mAbs in the healthcare settings (Table 1). Among the best examples for the SARS-CoV-2-specific mAb therapeutics is the NCT04425629. This is composed of an anti-Spike (S) SARS-CoV-2 mAb cocktail, combining a fraction of spike antibody from a person who recently recovered and one fraction from a mouse immunized with the SARS-CoV-2-S protein. For the preparation of the NCT04425629 mAbs, a large antibody panel against the S protein was prepared from humanized mice and from COVID-19 recovered patients. From this panel, the antibody pair, REGN10987 and REGN10933, was chosen based on its different target epitopes. While REGN10933 binds at the top of the RBD, REGN10987 targets an epitope located on the side of the RBD, indicating no competition for binding to the RBD. The patient group involved in the NCT04425629 was selected based on several primary outcomes, including patients with treatment-emergent serious adverse events, patients with infusion-related reactions, and patients with hypersensitivity reactions. In a recent work, Baum et al. reported protective effects of the REGN10987/REGN10933 mAb cocktail in rhesus macaques and golden hamsters. The mAb cocktail reduced virus load and virus-induced pathological sequelae in rhesus macaques. In addition, the cocktail resulted in limited weight loss and evidence of pneumonia in hamsters [57]. This cocktail is currently involved in three large-scale, placebo-controlled trials to evaluate its safety, tolerability, and efficacy for the treatment of COVID-19. In a recent study, two ultra-potent SARS-CoV-2 human mAbs (S2E12 and S2M11), which could provide additional benefits for clinical application, were isolated and characterized by Tortorici et al. [58]. Among approximately 800 screened mAbs isolated from individuals who recovered from COVID-19, the two mAbs were chosen based on their protective effect against SARS-CoV-2 challenge in hamsters [58].

### 4.2. SARS-CoV-2 Immunomodulatory Antibody-Based Therapeutics

As mentioned above, SARS-CoV-2 infections resulted in dysregulation of the immune response as well as the cytokine storm, due to the overproduction of inflammatory cytokines and chemokines, with impaired innate protection mechanisms such as the type-1 IFN response, are mainly responsible for the immunopathology in severe COVID-19 cases [33,45,59,60,61,62,63]. Based on these facts, several clinical trials are currently evaluating different immunotherapeutic approaches using the immunomodulatory monoclonal antibodies, which target the pathways triggered by SARS-CoV-2 infection, aiming to control the immune response and the inhibition of the cytokine storm in COVID-19 patients. This includes mAb targeting inflammatory mediators, such as IL-6, IL-1β, IL-2, IL-8, IL-17, G-CSF, GM-CSF, IP10, MCP1α, MIP1β, TNFα, and complement component-5 [64,65,66,67,68].

In the case of COVID-19 patients with severe pneumonia and ARDS, elevated serum levels of the inflammatory cytokine IL-6 were a marker for poor outcomes. Several clinical trials (NCT04317092, NCT04320615, and NCT04370834) are currently under evaluation in many countries (Italy, USA, and China) to evaluate the efficacy, safety, pharmacodynamics, and pharmacokinetics of the IL-6 receptor-targeted mAb tocilizumab to dampen the inflammatory response in patients with severe COVID-19 [69,70,71,72,73]. Another potential target of COVID-19 immunomodulatory mAb therapy is the inflammatory cytokine IL-1β, which plays an essential role in the cytokine storm associated with SARS-CoV-2 infection [74]. A promising therapeutic inhibition for the IL-1β is Canakinumab, which has been previously used effectively in the treatment of different inflammatory syndromes [67,68]. Canakinumab is a high affinity human mAb against interleukin IL-1β, which inhibits the pro-inflammatory effects of IL-1β by blocking its binding to IL-1R. Several clinical trials (NCT04365153, NCT04348448) have begun to evaluate the use of Canakinumab for the treatment of COVID-19 [67,68]. Increasing evidence suggests the contributions of the T-helper 17 cell cytokine IL-17 to COVID-19-related ARDS [75,76,77]. Therefore, targeting the IL-17 signaling using anti-IL-17 mAbs is currently under investigation for the treatment of COVID-19 patients, particularly those with ARDS [76]. The human IL-17-specific mAb, Secukinumab, is currently under a phase II clinical trial (NCT04403243) for the treatment of COVID-19 patients [78]. In addition to inflammatory cytokines, complement activation also contributes to the pathology of severe COVID-19 cases. The complement activation is an innate immune mechanism to pathogens with an essential role in pro-inflammatory immune responses [79]. Therefore, an ongoing clinical trial (NCT04371367) is evaluating the therapeutic effect of inhibiting the complement activation during the early stage of SARS-CoV-2-infection using a mAb to the complement component-5a receptor 1 (CD88) [80]. In a recent randomized Phase 2 clinical trial, treatment with a blocking mAb against the complement protein C5a (vilobelimab) showed a mortality-reducing effect in patients with severe COVID-19 [23]. Based on this effect, the clinical evaluation of vilobelimab in a Phase 3 trial has been suggested by the authors [23].

Other therapeutic approaches for the COVID-19 include the targeting of some key signal pathways involved in the myeloid cell production, function, and maturation. Granulocyte-macrophage colony-stimulating factor (GM-CSF) binding to its α-receptor activates the secretion of multiple pro-inflammatory cytokines by the macrophages and the neutrophils, which affect their activation and differentiation [81]. Based on these facts, several clinical trials are currently taking place in the USA. These trials aim to evaluate the effectivity of different mAb against the human GM-CSF (NCT04351243, NCT04341116) or the GM-CSF receptor (NCT04447469, NCT04397497) for the treatment of COVID-19 [82,83,84]. 

To reduce the extravasation of blood neutrophils and monocytes and to avoid their accumulation in the lung and the collateral tissue damage to the airway epithelial cells and vascular endothelial cells of COVID-19 patients, additional clinical trials are progressing to exploring the use of mAb targeting the adhesion molecules and chemokine receptors [85]. A recent clinical trial (NCT04435184) is currently evaluating the use of Crizanlizumab 56], a mAb to P-selectin (a cell adhesion molecule expressed on the endothelial cells of blood vessels and activated platelets) for treatment of COVID-19 [86,87]. This approach is designed to inhibit the extravasation and recruitment of inflammatory cells to the lungs. Other trials (NCT04343651 and NCT04347239) involve targeting the process of the recruitment of monocytes and neutrophils by blocking the CC chemokine receptor-5 (CCR5; CD195) using mAbs [88].

Toll-like receptors (TLR) play critical roles in the initiation of the inflammatory response [89,90]. The use of mAb to the LPS-receptor CD14 (NCT04391309) for damping the hyperactivation of innate immune cells in COVID-19 patients is currently under evaluation [91]. Another clinical trial (NCT04317040) is also exploring the employment of CD24Fc, a recombinant fusion protein consisting of the CD24 extracellular domain and IgG1-Fc domain, which binds to danger-associated molecular patterns (DAMPs) released from injured cells, thereby blocking the sensing of DAMPs through PRRs and inhibiting the secretion of inflammatory cytokines [92]. Further clinical trials are currently evaluating additional immunomodulatory antibody-based therapeutics. This includes mAb to the immunomodulatory cytokine IFNγ (NCT04324021), the connective tissue growth factor (NCT04432298), the vascular endothelial growth factor (NCT04305106), and the T cell surface molecule CD147 (NCT04275245), which has recently been shown to bind to SARS-CoV-2-S protein and contribute to the lymphopenia reported in COVID-19 patients [37,93,94,95,96,97].

## 5. Overview and Analysis of the Current Clinical Trials

The current clinical trials of using monoclonal antibodies for treating Covid-19 are summarized in Table 1. The table summarizes the data concerning (1) the clinical trials’ IDs, (2) the countries in which the clinical trials are held, (3) the recruiting status and the number of recruitments, (4) the sponsorship of the clinical trial projects, and (5) the concept used in trial design. The dataset was tabulated and prepared for the statistical analysis using STATA statistical software.

The “Estimated Enrollment” variable had a continuous nature, so we drew a box plot to check the median estimated enrollment and the spread of the data. All other variables had a qualitative nature, which were then categorized by the STATA software. For instance, we considered the country variable to be coded as 1, 2, 3, 4, etc. Similarly, all the other variables were categorized and coded according to their characteristics and levels. The purpose of coding is to generate frequency and percentage tables using statistical software (Table 2).

We performed descriptive analysis and data visualization for the variables related to the clinical trials in the study. The associations of different indicators with each other were tested to evaluate whether the indicators are interlinked or not. A cross-tabulation analysis was used to check the frequencies, and chi-square was used to test the association between them. If the chi-square test statistics value was significant, then we concluded that there was a significant association between the two variables. In our analysis, we used alpha significance equal to 5%.

The descriptive statistics of “estimated enrollments” are provided in Table 3. The total estimated enrollments in all trials were 15,147, with an average of 489 per trial location with minimal recruitments of 20, and the maximal number was 2924. 

**Figure 2:**
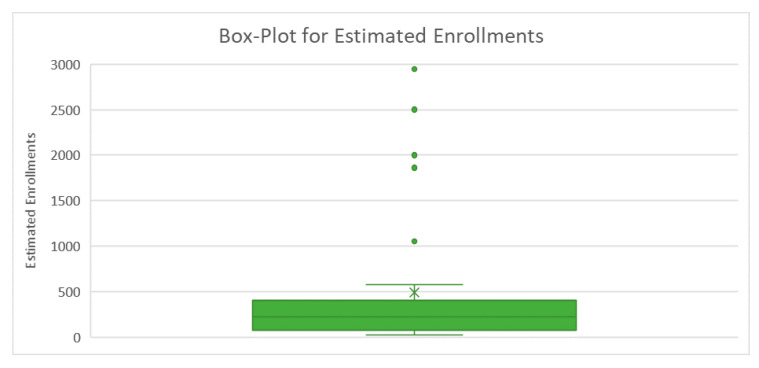
Box plot of the estimated enrollments during COVID-19 clinical trials.

A boxplot was created to obtain a clear picture of the information inside the estimated enrollment (Figure 2). The boxplot comprises the minimum value, maximum value, Quartile 1, Quartile 2, and median of the variable. The median value for the estimated enrollments was 220, with some extreme values. In addition, most of the observed numbers were in the range of 20 and 450.

To gain more insight into the clinical trials characteristics, a cross-tabulation was undertaken between the percent of trial contribution by the country in which the trial was held and sponsoring institutions (Table 4). The highest sponsorship rates were observed by hospitals and medical colleges (45.16%) followed by pharmaceutical companies (32.26%). The sponsorship from biopharmaceutical companies was the highest in the USA (22.58%), compared with 6.46% in Italy and 3.23% in China. In contrast, the sponsorship from hospitals and the medical colleges was the most predominant in China, compared with other countries. There was a significant association between the country and sponsorship (*p* < 0.05). Moreover, the total percentage of sponsoring projects was the highest in USA (58.07%) followed by China (22.5%), Italy (16.15%), and France (3.23%).

**Figure 3:**
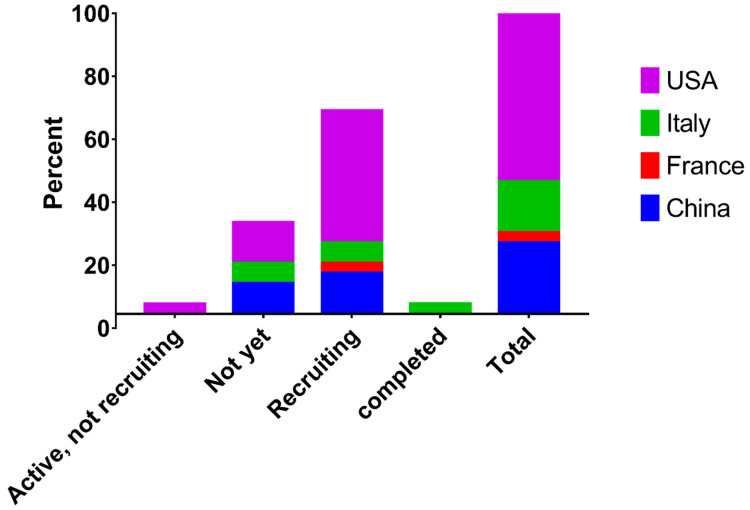
The percent of trial recruitments status by the country in which the trial was held. USA has a higher percentage of recruited people (41.945) followed by China, Italy, and France.

There was no significant statistical association between the phases of clinical trials or the status of recruitment and the country in which the clinical trial is held. Since the largest number of trials was in the USA, there was a corresponding higher number of recruiting status as well as the largest non-recruiting rates (Table 5, Figure 3).

## 6. Conclusions and Future Perspectives

The short-term immunity induced by using antibody-based immunotherapeutics represents an effective alternative strategy to bridge the gap resulting from the lack of an efficient vaccine against SARS-CoV-2. In addition to their proven record of safety and efficacy, antibody-based immunotherapeutics have shorter development and testing timeline compared to vaccines or other chemical drugs [50,98]. The current COVID-19 antibody-based immunotherapeutic approaches include SARS-CoV-2-neutralizing antibodies, such as convalescent plasma, NAbs, MAbs, and IVIg, and immunomodulatory antibodies [54,64,65,66,67,68]. Although the passive administration of convalescent plasma represents a possible treatment for critical COVID-19 patients, it must overcome several challenges, including the existence of non-neutralizing or subneutralizing antibodies, which may result in antibody-dependent enhancement of viral infection. Separated SARS-CoV-2-specific neutralizing antibodies and newly developed SARS-CoV-2-specific monoclonal antibodies are viable options. Several animal model studies have reported different dysregulating effects of S-protein-specific IgG antibodies, including the polarization of T helper cells toward a Th2 response with increased production of type 2 cytokines resulting in severe acute diffuse pulmonary alveolar damage in the lungs [53,99]. As the development of new effective antiviral immunomodulatory mAbs is a time-consuming process, several clinical trials are currently evaluating the repurposing of existing immunomodulatory mAbs to modulate the host immune defense against SARS-CoV-2 infection as well as to avoid the harmful overreaction of the immune system [53,99].

The immune response plays vital roles during the course of SARS-CoV-2 infections in most patients. Understanding different aspects of the molecular immunology of SARS-CoV-2 should open new avenues for the intervention of the treatment, especially in severely affected patients. Application of the antibody-based immunotherapeutic strategies showed promising trends in the treatment of some types of cancers and viral infections in the past. In light of our understandings of SARS-CoV-2 and its immune evasion and manipulation strategies, we assume the application of the antibody-based immunotherapeutic, especially the monoclonal antibodies, will have a great impact on the treatment of severely affected cases of SAR-CoV-2. This will reduce the overall number of hospitalizations and will dramatically decrease the case fatality rate among severe cases, which have a bad prognosis. More research is urgently needed to understand various immune evasion strategies of SARS-CoV-2 together with the various studying aspects of the SARS-CoV-2 host interaction.

## Figures and Tables

**Table 1:** The current clinical trials of using monoclonal antibodies for treating COVID-19.

ID	Recruiting	Country	Sponsor	Study Design	Estimated Enrollment	Intervention	Concept	Phase
NCT04261426	Not yet	China	Medical college hospital	Single-center, randomized, open-label, controlled trial	80	Intravenous immunoglobulin therapy	Providing passive immunity and anti-inflammatory, immunomodulatory effect.	Phase 2/Phase 3
NCT04268537	Not yet	China	University	Randomized, parallel assessment	120	PD-1 blocking antibody	Evaluating the efficacy of the Programmed cell death (PD)-1 and thymosin in COVID-19 patients with severe pneumonia associated with lymphocytopenia	Phase 2
NCT04275245	Recruiting	China	Hospital	Single-group, randomized, open-label, trial	20	Meplazumab	Humanized anti-CD147 antibody	Phase 1/Phase 2
NCT04293887	Not yet	China	Medical college hospital	Randomized, Open label, parallel assessment	328	Recombinant human IFN-α2β	Efficacy and safety of IFN-α2β	Early Phase 1
NCT04305106	Recruiting	China	University hospital	Multicenter Randomized Controlled Clinical Trial	140	Bevacizumab	Antibody against vascular endothelial growth factor (VEGF), which is known as the most potent inducing factors to increase vascular permeability	
NCT04315298	Recruiting	USA	Multicenter sponsored by pharmaceutical companies	Multicenter Randomized parallel assessment Clinical Trial	2500	Sarilumab	mAb targeting IL-6R	Phase 2/Phase 3
NCT04317040	Recruiting	USA	Multicenter sponsored by pharmaceutical company	Multicenter Randomized parallel assessment Clinical Trial	230	CD24Fc	Investigating the immunomodulatory effect of CD24Fc in COVID-19 treatment	Phase 3
NCT04317092	Recruiting	Italy	National institute	Open label single group assessment	400	Tocilizumab	IL-6 inhibitor	Phase 2
NCT04320238	Recruiting	China	University hospital	Nonrandomized open-label, parallel assessment Clinical Trial	2944	rhIFNα	Nasal Drops of recombinant hIFNα to prevent COVID-19 in medical staff	Phase 3
NCT04320615	Active, not recruiting	USA	Multicenter sponsored by pharmaceutical companies	Multicenter Randomized parallel assessment Clinical Trial	450	Tocilizumab	evaluate the efficacy, safety, pharmacodynamics, and pharmacokinetics of tocilizumab, IL-6 inhibitor	Phase 3
NCT04322188	completed	Italy	Hospital	Observational, retrospective study	220	Siltuximab	IL-6 inhibitor used for cancer therapy	
NCT04324021	Recruiting	Italy	Biopharmaceutical company	Multicenter Randomized parallel assessment Clinical Trial	54	Emapalumab, Anakinra	A combination of an anti-IFNγ mAb (Emapalumab) and an IL-1 receptor antagonist (Anakinra)	Phase 2/Phase 3
NCT04441918	Recruiting	China	Biopharmaceutical company	Randomized open label, Clinical Trial	40	JS016	Investigating the Safety, Tolerability, Pharmacokinetics, and immunogenicity of a recombinant humanized Anti-SARS-CoV-2 mAb (JS016)	Phase 1
NCT04426695	Recruiting	USA	Multicenter sponsored by pharmaceutical companies	Multicenter Randomized parallel assessment Clinical Trial	1860	Anti-Spike antibody	mAb against S Protein of SARS-CoV-2	Phase 1/Phase 2/Phase 3
NCT04425629	Recruiting	USA	Multicenter sponsored by pharmaceutical companies	Multicenter Randomized parallel assessment Clinical Trial	1054	REGN10933 + REGN10987 antibody cocktail	Evaluating the Safety, Tolerability, and efficacy of mAb to SARS-CoV-2 S Protein for the treatment of ambulatory patients with COVID-19	Phase 1/Phase 2/Phase 3
NCT04351152	Recruiting	USA	Multicenter sponsored by pharmaceutical companies	Multicenter Randomized parallel assessment Clinical Trial	238	Lenzilumab	For cytokine release syndrome mediated hyper-immune reaction (“cytokine storm”)	Phase 3
NCT04371367	Recruiting	France	Hospital, sponsored by pharmaceutical companies	Randomized parallel assessment Clinical Trial	108	Anti-C5aR	Complement component 5a receptor 1 or CD88 is a G protein-coupled receptor for C5a that regulates inflammation	Phase 2
NCT04391309	Not yet	USA	Hospital, sponsored by pharmaceutical companies	Randomized parallel assessment Clinical Trial	300	IC14	IC14, monoclonal antibody to CD14	Phase 2
NCT04429529	Recruiting	USA	sponsored by pharmaceutical companies	Randomized parallel assessment Clinical Trial	25	TY027	Anti-SARS CoV2 antibody	Phase 1
NCT04447469	Recruiting	USA	sponsored by pharmaceutical companies	Randomized parallel assessment Clinical Trial	573	Mavrilimumab (KPL-301)	Mavrilimumab is a human mAb that inhibits human GM-CSF-R ()	Phase 2/Phase 3
NCT04370834	Recruiting	USA	sponsored by pharmaceutical companies	Single group assignment	219	Tocilizumab	Using IL-6 mAb for treatment of COVID-19 patients with Cancer	Phase 2
NCT04351243	Recruiting	USA	Hospital, sponsored by pharmaceutical companies	Multicenter Randomized parallel assessment Clinical Trial	270	Gimsilumab	Gimsilumab acts on GM-CSF	Phase 2
NCT04365153	Recruiting	USA	Hospital, sponsored by pharmaceutical companies	Randomized, factorial assessment	45	Canakinumab	Canakinumab is antibody targeting interleukin-1 beta	Phase 2
NCT04348448	Not yet	Italy	sponsored by pharmaceutical companies	Retrospective and prospective observational study	100	Canakinumab	Canakinumab is antibody targeting interleukin-1 beta	
NCT04343651	Not yet	USA	sponsored by pharmaceutical companies	Randomized parallel assessment Clinical Trial	75	Leronlimab	Anti CC chemokine receptor 5 (CCR5; CD195) antibody	Phase 2
NCT04452318	Not yet	USA	sponsored by pharmaceutical companies	Randomized parallel assessment Clinical Trial	2000	REGN10933 + REGN10987	mAb against the S Protein of SARS CoV-2	Phase 3
NCT04432298	Recruiting	USA	Hospital, sponsored by pharmaceutical companies	Randomized parallel assessment Clinical Trial	130	Pamrevlumab	Pamrevlumab against connective tissue growth factor (CTGF)	Phase 3
NCT04341116	Recruiting	USA	sponsored by pharmaceutical companies	Randomized parallel assessment Clinical Trial	144	TJ003234	TJ003234 is an antibody against human GM-CSF	Phase 1/Phase 2
NCT04347239	Recruiting	USA	sponsored by pharmaceutical companies	Randomized parallel assessment Clinical Trial	390	Leronlimab	antibody against CC chemokine receptor 5 (CCR5; CD195)	Phase 2b/Phase 3
NCT04397497	Not yet	Italy	hospital	Randomized parallel assessment Clinical Trial	50	Mavrilimumab	Inhibits human GM-CSF-R	Phase 2
NCT04435184	Not yet	USA	Hospital, sponsored by pharmaceutical companies	Randomized parallel assessment Clinical Trial	40	Crizanlizumab	Active against P-selctin, a cell adhesion molecule on the surfaces of activated endothelial cells, which line the inner surface of blood vessels, and activated platelets. Prevent vaso-occulsive crises.	Phase 2

The table summarizes the data concerning (1) the clinical trials’ IDs, (2) the countries in which the clinical trials are held, (3) the recruiting status and number of recruitments, (4) the sponsorship of the clinical trial projects, and (5) the concept used in trial design. The dataset was tabulated and prepared for the statistical analysis using STATA statistical software.

**Table 2:** The variables used in the study and their description.

Variables	Description
ID	Qualitative
Recruiting	Qualitative
Country	Qualitative
Sponsor	Qualitative
Study design	Qualitative
Estimated enrollment	Quantitative
Intervention	Qualitative
Concept	Qualitative
Phase	Qualitative

**Table 3:** Summary Statistics for the estimated enrollment.

Parameter	Value
Mean	489
Standard Error	137
Median	219
Mode	40
Standard Deviation	764
Sample Variance	584,098
Kurtosis	4
Skewness	2
Range	2924
Minimum	20
Maximum	2944
Sum	15,147
Count	31

**Table 4:** Cross-tabulation between the percent of trial contribution by the country in which the trial was held and the sponsoring institutions.

Sponsor	Country				
	China	France	Italy	USA	Total
Biopharmaceutical companies	3.23	0	6.46	22.58	32.26
Hospital, medical college, or university hospital	19.36	3.23	6.46	16.13	45.16
Multicenter sponsored	0.00	0.00	0.00	19.36	19.36
National institute	0.00	0.00	3.23	0.00	3.23
Total	22.59	3.23	16.15	58.07	100.00

Pearson chi2(30) = 48.1710; Pr = 0.019.

**Table 5:** Cross-tabulation between the percent of trial recruitments status by the country in which the trial was held.

Recruiting Status	Country				
	China	France	Italy	USA	Total
Active, not recruiting	0.00	0.00	0.00	3.23	3.23
Not yet	9.68	0.00	6.45	12.90	29.03
Recruiting	12.90	3.23	6.45	41.94	64.52
completed	0.00	0.00	3.23	0.00	3.23
Total	22.58	3.23	16.13	58.06	100

Pearson chi^2^(9) = 8.0537; Pr = 0.529.
